# The Effect of Endogenous Cushing Syndrome on All-cause and Cause-specific Mortality

**DOI:** 10.1210/clinem/dgac265

**Published:** 2022-04-29

**Authors:** Padiporn Limumpornpetch, Ann W Morgan, Ana Tiganescu, Paul D Baxter, Victoria Nyawira Nyaga, Mar Pujades-Rodriguez, Paul M Stewart

**Affiliations:** School of Medicine, University of Leeds, Leeds LS2 9NL, UK; Division of Endocrinology and Metabolism, Department of Internal Medicine, Faculty of Medicine, Prince of Songkla University, Hatyai, Songkhla 90110, Thailand; School of Medicine, University of Leeds, Leeds LS2 9NL, UK; NIHR Leeds Biomedical Research Centre, Leeds Teaching Hospitals NHS Trust, Leeds, UK; NIHR Leeds Medtech and In-vitro Diagnostics Co-operative, Leeds Teaching Hospitals NHS Trust, Leeds, UK; School of Medicine, University of Leeds, Leeds LS2 9NL, UK; School of Medicine, University of Leeds, Leeds LS2 9NL, UK; Unit of Cancer Epidemiology–Belgian Cancer Centre, Sciensano, Brussels, Belgium; School of Medicine, University of Leeds, Leeds LS2 9NL, UK; School of Medicine, University of Leeds, Leeds LS2 9NL, UK; NIHR Leeds Biomedical Research Centre, Leeds Teaching Hospitals NHS Trust, Leeds, UK

**Keywords:** Cushing syndrome, mortality, meta-analysis, causes of death, meta-regression analysis

## Abstract

**Objective:**

We aimed to perform a systematic review and meta-analysis of all-cause and cause-specific mortality of patients with benign endogenous Cushing syndrome (CS).

**Methods:**

The protocol was registered in PROSPERO (CRD42017067530). PubMed, EMBASE, CINHAL, Web of Science, and Cochrane Central searches were undertaken from inception to January 2021. Outcomes were the standardized mortality ratio (SMR), proportion, and cause of deaths. The *I*^2^ test, subgroup analysis, and meta-regression were used to assess heterogeneity across studies.

**Results:**

SMR was reported in 14 articles including 3691 patients (13 Cushing disease [CD] and 7 adrenal CS [ACS] cohorts). Overall SMR was 3.0 (95% CI, 2.3-3.9; *I*^2^ = 80.5%) for all CS, 2.8 (95% CI, 2.1-3.7; *I*^2^ = 81.2%) for CD and 3.3 (95% CI, 0.5-6.6; *I*^2^ = 77.9%) for ACS. Proportion of deaths, reported in 87 articles including 19 181 CS patients (53 CD, 24 ACS, and 20 combined CS cohorts), was 0.05 (95% CI, 0.03-0.06) for all CS subtypes with meta-regression analysis revealing no differences between CS subtypes (*P* = .052). The proportion of deaths was 0.1 (10%) in articles published before 2000 and 0.03 (3%) in 2000 until the last search for CS (*P* < .001), CD (*P* < .001), and ACS (*P* = .01). The causes of death were atherosclerotic diseases and thromboembolism (43.4%), infection (12.7%), malignancy (10.6%), active disease (3.5%), adrenal insufficiency (3.0%), and suicide (2.2%). Despite improved outcomes in recent years, increased mortality from CS persists. The causes of death highlight the need to prevent and manage comorbidities in addition to treating hypercortisolism.

Endogenous Cushing syndrome (CS) refers to inappropriate hypercortisolism caused by either adrenocorticotropin (ACTH) hypersecretion or autonomous adrenal cortisol hypersecretion (1, 2). These are rare diseases with limited epidemiological data, with publications largely restricted to Cushing disease (CD) (3, 4). CD is the most common subtype accounting for 70%, followed by adrenal CS (ACS) 20% to 25%, and ectopic CS 5% to 10% (5). The overall incidence of all-cause CS is estimated to range between 1.8 and 3.2 cases per million per year (6, 7), between 0.6 and 2.6 per million per year for CD (3, 4, 6-8), between 0.3 and 0.7 per million per year for benign ACS (6, 7, 9, 10), 0.2 per million per year for adrenocortical carcinoma, and 0.8 per million per year for ectopic CS (3, 6, 11).

In recent decades, numerous advances have led to greater understanding of CS. Our improved ability to diagnose and provide multimodality therapies offer the opportunity to achieve a “cure.” Some studies of all-cause and cause-specific mortality associated with endogenous CS have reported an increased risk of death (3, 9, 12–18), but due to the low disease incidence, single CS studies have insufficient power to reliably estimate patient mortality. A previous systematic review and meta-analysis of CS dating back to 2012 reported on 797 CS patients drawn from 7 publications. The majority of patients had CD (n = 688) with a standardized mortality ratio (SMR) of 1.8 (95% CI, 1.3-2.7). A smaller proportion had adrenal adenoma (AA; n = 109) with an SMR of 1.9 (95% CI, 0.9-3.9). This meta-analysis did not find a statistically significant difference in surgically cured CD (SMR of 1.2; 95% CI, 0.5-3.0) or AA mortality over and above the general population (17). To date, no published systematic review or meta-analysis has assessed the overall mortality and specific causes of death across all the different subtypes of CS. In an attempt to better define our current understanding of patient outcomes and improve future treatments, this meta-analysis aimed to evaluate the SMR and the proportion of deaths in patients with benign endogenous CS and its subtypes and the causes of death.

## Materials and Methods

This study was conducted according to the principles of the Preferred Reporting Items for Systematic Reviews and Meta-analysis (PRISMA) (19, 20) and was registered in the International Prospective Register of Systematic Reviews (PROSPERO; CRD42017067530). All protocol steps were performed by 2 authors (P.L. and M.P.R.) and verified by author P.M.S. Discrepancies were resolved through team discussion between authors P.L., M.P.R., A.W.M., and P.M.S.

### Search Strategy

The literature search from 1945 to January 31, 2021 was performed on PubMed/MEDLINE, Cochrane Library, EMBASE, Web of Science, and CINAHL. A combination of well-defined key search terms for Cushing syndrome (Cushing*, adrenal tumour*, adrenal adenoma*, adrenocortical adenoma*, glucocorticoid producing adenoma*, glucocorticoid producing tumour*, Cushing disease, ACTH producing tumour*, ACTH-secreting tumour*, ACTH-producing adenoma*, pituitary tumour*) and terms for the study outcome (death and mortality) were used. The reference lists of eligible articles or relevant systematic reviews of CS were also screened to identify other potentially eligible studies.

### Selection Criteria

The reviewed included articles reporting estimates of SMRs or the number of deaths from cohorts of adults aged 18 years with endogenous CS. To minimize the risk of selection bias (21), studies included had a minimum sample size of 10 participants. Exclusion criteria were: 1) non-English publications; 2) nonhuman studies; 3) case reports, case series, conference abstracts, book chapters, systematic reviews or clinical guidelines; 4) studies of exogenous CS; 5) those conducted among high-mortality populations (ie, adrenal cell carcinoma, malignancy pituitary tumors, or ectopic CS); and 6) articles solely reporting on the long-term follow-up of patients with CS in remission. To avoid duplications, the most recently published or larger and longer-term study was selected among those based on the same data sources.

### Outcome Definitions

The primary outcomes were the SMR and the proportion of deaths from all-cause mortality reported at the longest duration of follow-up. Secondary outcomes were the SMR and the number of deaths from specific causes.

### Review Procedures and Data Extraction

The articles identified from every search engine were deduplicated using Endnote version X9 and Rayyan software programs (22). Potential duplications were identified by comparing articles reporting on the same diseases form the same centers and assessing overlap in years of follow-up covered. The European cohorts, including the European Cushing’s Disease Survey Group (23) and ERCUSYN (24), were also assessed, and 11 duplicated articles were excluded on this basis (11-13, 25-32). Covidence software was used for the title, abstract, and full-text screening (33).

Data extraction forms were designed and implemented using Microsoft ACCESS version Office 365. The following data were extracted: PubMed identification, first author, country, hospital and publication year, study design, sample size, age, sex, CS subtype (CD, ACS, combined), CD subtypes (microadenoma, macroadenoma), disease activity (active, in remission), ACS subtypes (adrenal adenoma [AA], bilateral adrenal hyperplasia [BAH]), level of care (community, primary, secondary or tertiary care), data source and period of recruitment or observation, treatment, timing of reported mortality (perioperative period [deaths occurring ≤ 30 days of a surgical procedure]) or long-term follow-up (deaths that occurring > 30 days after the operation), follow-up period, and mortality (overall, cause-specific SMR, number of deaths). We contacted the authors to obtain missing data. Extracted data were reviewed and cross-checked against the electronic records by P.L., M.P.R., and P.M.S.

### Quality Assessment

The risk of bias of all included studies was independently evaluated through a modified version of the ROBIN-I tool (34) by P.L. and M.P.R. The modified tool consisted of 7 bias domains: confounding and selection bias, classification and diagnosis of CS, deviations from intended interventions, missing data, mortality ascertainment, and reporting bias. The risk of bias at the domain level and overall in each study was classified as low, moderate, serious, critical, and uncertain (ie, no information on which to base a judgment) (34).

### Data Synthesis, Meta-Analysis, Meta-Regression, and Subgroup Analysis

The SMR represented the number of CS deaths in the study compared to the expected number of deaths in an age- and sex-matched normal population. Random-effects meta-analysis of SMRs was conducted, using the inverse-variance weighting of log-SMR from each study to calculate summary estimates and produce forest plots by exponentiating the log-SMR.

The estimated proportion of deaths was obtained by meta-regression under the assumption of the binomial distribution, using mixed-effects logistic regression. Between-study heterogeneity was expressed as the *I*^2^ value with *I*^2^ values of less than 25% representing low level, 25% to 49% moderate level, 50% to 74% substantial level, and more than 75% indicating high level (35). We explored sources of heterogeneity with meta-regression and subgroup analyses by (1) CS subtypes: CD, ACS, and combined types; (2) CD activity: active and remission; (3) pituitary size: microadenoma vs macroadenoma; (4) perioperative and longer-term mortality; (5) study period (or published time) to account for diagnosis, treatment, and care practices; and (6) operative procedures (transsphenoidal surgery, adrenalectomy). Causes of death data were extracted when available and classified. All statistical analyses were performed using STATA version 16.1 (Stata Corp) (36) with “metan” (37) and “metapreg” (38) packages.

Sensitivity analyses were conducted for the primary outcome by excluding studies with critical, serious, uncertain, or moderately biased articles.

## Results

**Figure 1.**
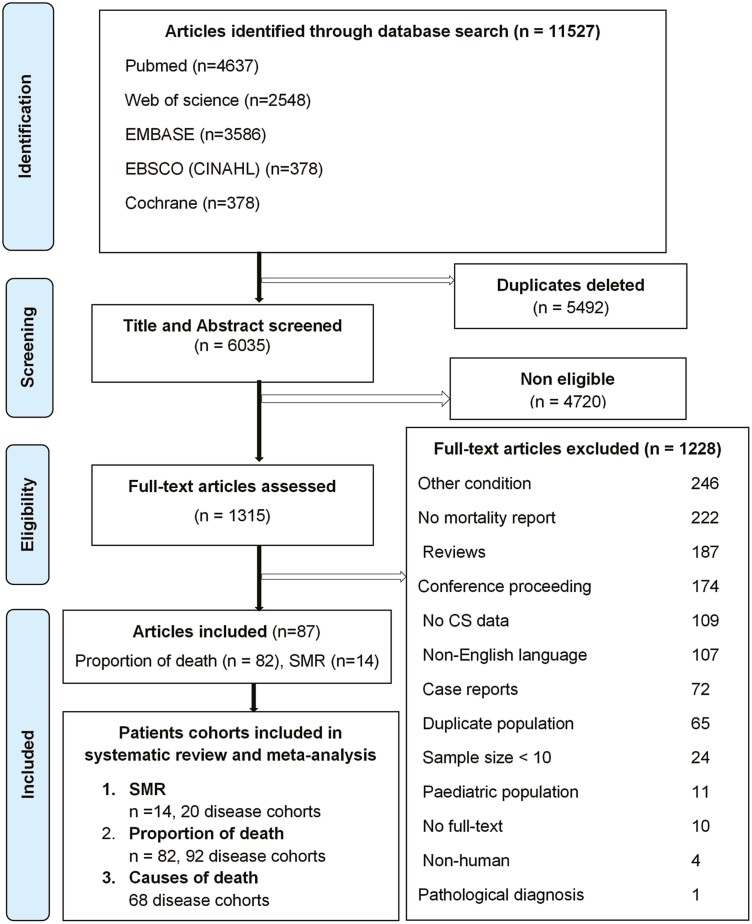
PRISMA flow diagram detailing articles and cohorts included for systematic review and meta-analysis. CS, Cushing syndrome; PRISMA, Preferred Reporting Items for Systematic Review and Meta-Analysis Protocols; SMR, standardized mortality ratio.

Fig. 1 summarizes the PRISMA study flow diagram. Of 11 527 articles identified in the initial search, 92 articles were eligible for inclusion. The SMR analyses encompassed 14 articles reporting on 20 cohorts including 3691 patients. Eighty-two articles described 92 cohorts containing 19 181 CS patients, reporting the number (or proportion) of deaths. Five articles were considered in SMR analyses but omitted from analyses of the proportion of deaths to prevent duplication (6, 13, 14, 39, 40). Table 1 describes individualized characterization of the underpinning research articles reporting SMR. Of 20 SMR cohorts, 13 were CD cohorts comprising 2160 patients and 7 ACS cohorts with 1531 patients. Articles reporting SMR for specific subpopulations included active CD (n = 262) (6, 8, 9, 12, 14, 16), CD in remission (n = 1234) (6, 8, 9, 12, 14, 16, 41), microadenoma (n = 332) (12, 39), macroadenoma (n = 60) (9, 12), AA (n = 158) (6, 9, 13), and BAH (n = 20) (9, 13). Concerning causes of death, 4 cohorts reported SMR for ischemic cardiovascular diseases (CVDs), and 2 for infection.

**Table 1.** Characteristics of articles reporting estimates of standardized mortality ratios in Cushing syndrome

Study	Country	Obs period	Age^*a*^, y	No. CS	No. death	Follow-up^*a*^,	CS subtypes	SMR (95% CI)
**CD**								
Etxabe, 1994 (3)	Spain (multicenter)	1975-1992	39.6	49	5	6.6 (4.7)	Unknown 100%	3.8 (2.5-17.9)^*c*^
Pikkarainen, 1999 (42)	Finland (single center)	1981-1996	44.6	44	8	NR	Micro 86.4%, macro 11.4%, unknown 2.3%	2.7 (0.9-5.3)^*d*^
Swearingen, 1999 (39)	US (single center)	1978-1996	38 (38)	161	6	8.7 (8.0)	Micro 100%	1.0 (0.4-2.2)^*e*^
Lindholm, 2001 (6)	Denmark (nationwide)	1985-1995	(41.1)	73	7	(8.1)	Unknown 100%	1.7 (0.7-3.5), proven^*f*^
			(51.1)	26	11	(8.1)		Unproven: 11.5 (5.7-20.5)
			(38.5)	45	1	(9.1)		Remission: 0.3 (0.01-1.7)
			(46.4)	20	6	(10.0)		Active: 5.1 (1.9-11.0)
Hammer, 2004 (40)	US (single center)	1975-1998	(37.0)	289	25	(11.1)	Micro 48.4%, macro 20.8%, unknown 30.8%	1.4 (1.0-2.1)^*g*^
			(37.0)	236	17	(11.1)		Remission: 1.2 (0.7-3.4)
			(37.0)	53	7	(11.1)		Active: 2.8 (1.4-11.0)
Dekkers, 2007 (14)	Netherlands (single center)	1977-2005	39.1	74	12	12.8	Micro 85.1%, macro 14.9%	2.4 (1.2-3.9)
			39.1	59	7	12.8		Remission: 1.8 (0.7-3.8)
			39.1	15	5	12.8		Active: 4.4 (1.4-9.1)
Bolland, 2011 (9)	New Zealand (nationwide)	1960-2005	39.0	188	24	NR	Micro 84.0%, macro16.0%	3.2 (2.6-3.8)^*h*^
			36.0	158	19	(7.5)		Micro: 3.2 (2.0-4.8))
			45.0	30	5	(6.9)		Macro: 3.5 (1.3-7.8)
			36.0	117	NR	(7.5)		Micro (remission): 3.1 (1.8-4.9)
			36.0	37	NR	(7)		Micro (active): 2.4 (0.4, 7.8)
			45.0	14	NR	(7.5)		Macro (remission): 2.5 (0.4-8.3)
			45.0	19	NR	(6.9)		Macro (active): 5.7 (1.4-8.3)
			36.0	158	19	(7.5)		Micro: 3.2 (2.0-4.8))
Clayton,2011 (16)	UK (single center)	1958-2010	(38.2)	60	13	(15.0)	Unknown 100%	4.8 (2.8-8.3)^*i*^
			(38.5)	54	8	(17.5)		Remission: 3.3 (1.7-6.7)
			(46.0)	6	5	(15.0)		Active: 16.0 (6.7-38.4)
Hassan-Smith, 2012 (41)	UK (single center)	1988-2009	(40.0)	80	13	(10.9)	Unknown 100%	3.2 (1.7-5.4)^*j*^
			(40.0)	52	5	(10.9)		Remission: 2.5 (0.8-5.8)
			(40.0)	20	4	(10.9)		Active: 16.0 (6.7-38.4)
Yaneva, 2013 (13)	Bulgaria (single center)	1965-2010	36.0	240	66	(8.8)	Unknown 100%	1.9 (0.7-4.1)^*k*^
Ntali, 2013 (12)	UK (single center)	1962-2009	(39.5)	182	26	(12.0)	Micro 87.4%, macro 12.6%	9.3 (6.2-13.4)^l^
			(39.5)	155	13	(12)		Remission: 10.8 (6.0-18.0)
			(39.5)	23	5	(12.0)		Active: 9.9 (3.6-21.9)
			(39.5)	155	19	(12.0)		Micro (remission): 7.6 (4.7-11.7)
			(39.5)	19	3	(12)		Micro (active): 6.5 (1.7-17.8)
			(39.5)	23	5	(12)		Macro: 15.6 (5.7-34.6)
			(39.5)	7	2	(5.0)		Macro (active): 45.5 (7.6-150.2)
Ragnarsson, 2019 (8)	Sweden (nationwide)	1987-2014	43.0	502	133	(13.0)	Unknown 100%	2.5 (2.1-2.9)^*m*^
			41.0	419	89	(15)		Remission: 1.9 (1.5-2.3)
			56.0	40	22	(4)		Active: 6.9 (4.3, 10.0)
Roldán-Sarmiento, 2021 (43)	Mexico (single center)	1979-2018	33.0	172	18	(7.5)	Micro 79.1%, macro 21.9%	3.1 (1.9-4.8)^*n*^
			33.0	83	8	(7.5)		Remission: 1.4 (0.6-2.6)
			33.0	29	8	(7.5)		active: 1.4 (0.6-32.6)
**ACS**								
Pikkarainen, 1999 (42) (combined)	Finland (single center)	1981-1997	NR	22	2	NR	AA 90.9%, BAH 9.1%	1.4 (0.2-4.9)
Lindholm, 2001 (6) (adenoma)	Denmark (nationwide)	1985-1995	(38.3)	37	4	(7.1)	AA 100%	3.5 (1.0-8.9)
Bolland, 2011 (9) (combined)	New Zealand (nationwide)	1960-2005	39.0	46	6	NR	AA 80.4%, BAH 19.6%	10.0 (5.8-14.1)
		1960-2005	41.0	37	3	(3.1)		AA 7.5 (1.9-20.0)
		1960-2005	41.0	9	3	(5.7)		BAH 14.0 (3.7-40.0)
Yaneva, 2013 (13) (adenoma)	Bulgaria (single center)	1965-2010	38.0	84	16	(4.2)	AA 100%	1.7 (0.2-6.0)
Yaneva, 2013 (13) (BAH)	Bulgaria (single center)	1965-2010	43.0	11	2	(5.5)	BAH 100%	1.1 (0.2-6.3)
Ntali, 2013 (12) (combined)	UK (single center)	1962-2009	(45.5)	16	1	(12.0)	Unknown 100%	5.3 (0.3-26.0)
Ahn, 2020 (10) (combined)	Korea (nationwide)	2002-2017	44.8	1127	74	(9.3)	AA 96.9%, BAH 3.1%	3.0 (2.4-3.7)^*o*^
**CS (combined CD and ACS)**, duplicated patients from the above data								
Pikkarainen, 1999 (42)	Finland (single center)	1981-1996	44.6	76	10	NR	Combined	2.0 (0.9-5.3)
Lindholm, 2001 (6)	Denmark (nationwide)	1985-1995	(41.4)	139	23	(8.1)	CD (proven) 52.5%, CD (unproven) 18.7%, ACS (AA) 28.8%	3.68 (2.3-5.3)
Bolland, 2011 (9)	New Zealand (nationwide)	1960-2005	39.0	234	36	(6.4)	CD 80.3%, AA 15.8%, BAH 3.9%	4.1 (2.9-5.6)
Yaneva, 2013 (13)	Bulgaria (single center)	1965-2010	38.0	335	84	(7.1)	CD 71.6%, AA 25.1%, BAH 3.3%	2.2 (1.1-4.1)

Abbreviations: AA, adrenal adenoma; ACS, adrenal Cushing syndrome; BAH, bilateral adrenal hyperplasia; CD, Cushing disease; CS, Cushing syndrome; macro, macroadenoma; micro, pituitary microadenoma; NR, not reported; Obs, observation; SMR, standardized mortality ratio; UK. United Kingdom; US, United States.

^*a*^Mean or (median) in years.

^*b*^

^*c*^Reference for expected numbers of deaths: age and sex group structures (Dirección de Información Sanitaria y Evaluación (1989) La mortalidad en la Comunidad Autónoma del País Vasco, 1987. Sistema Vasco de Información Sanitaria [SISVA], 6).

^*d*^Reference for expected numbers of deaths: Life tables for the expected mortality of the whole population for 1986 to 1990 were obtained from Statistics Finland.

^*e*^Reference for expected numbers of deaths: age- and sex adjusted sample of the US population.

^*f*^Reference for expected numbers of deaths: age- and sex specific mortality rates for Denmark 1991 to 1995.

^*g*^Reference for expected numbers of deaths: age and sex, divided into 5-year age groups, were obtained from the US Bureau of Census 1995, Monthly Vital Statistics Report 43.

^*h*^Reference for expected numbers of deaths: probability of each individual dying during follow-up using data from the Statistics New Zealand: New Zealand life tables (2000-2002) (http://www.stats.govt.nz).

^*i*^Reference for expected numbers of deaths: age, sex, and calendar year-specific mortality rates in the general population of England and Wales.

^*j*^Reference for expected numbers of deaths: age, sex, and calendar year-specific mortality rates in the general population of England and Wales.

^*k*^Reference for expected numbers of deaths (13): age and sex mortality rates in the Bulgarian general population (official data found at http://www.nsi.bg/otrasal.php?otrZ19).

^*l*^Reference for expected numbers of deaths: age, sex, and calendar year-specific mortality rates in the general population of England and Wales.

^*m*^Reference for expected numbers of deaths: general Swedish population for every calendar year and 5-year age group.

^*n*^Reference for expected numbers of deaths: age, sex, calendar year-specific mortality rates for the general population of England and Wales.

^*o*^Reference for expected numbers of deaths: age- and sex-matched 2015 Korean National Health and Nutrition Examination Survey (KNHANES).

Characteristics of studies reporting the proportion of deaths within the CS subtype cohorts are shown in Table 2. Forty-nine cohorts reported CD (14 971 patients), 24 ACS (2304 patients), including 7 AA cohorts, 2 BAH, cohorts and 15 combined ACS cohorts. Reports of pituitary tumor size in CD were established for 21%. Microadenoma was the majority of known pituitary size. ACS subtypes could be identified for 37.8%, of which AA were the majority of cases. Sixty-one cohorts, comprising 7148 patients, reported the causes of death.

**Table 2.** Patient characteristics of cohorts stratified by subtype of Cushing syndrome

Type	No. of studies	No. of patients (range)	Mean age at diagnosis (range), y	No. of women (%)^*a*^ (range)	Mean follow-up (range) (median follow-up) (range), y	No. of deaths (%)^*a*^
**All disease cohorts**	92	19 181 (13-5527)	40.9 (27.5-52.8), N = 68	7317 (60.5) (16-390), N = 65	6.4 (0.01-20), N = 36 (8.4) (0.01-15), N = 28	775 (4.0) (0-133)
**CD cohorts**	49	14 971 (18-5527)	40.4 (25.7-47.5), N = 34	3453 (59.4) (16-390), N = 41	5.8 (0.1-16.8), N = 21 (8.4) (1.4-15), N = 20	477 (3.2) (0-133)
**Pituitary microadenoma**	1	158	36	122	(7.5)	19
**Pituitary macroadenoma**	1	30	45	22	(6.9)	5
**Combined CD**	47	14 783 (18-5527)	40.5 (25.7-47.5), N = 32	3309 (59.4) (16-390), N = 39	5.8 (0.1-16.8), N = 21 (8.4) (1.4-15.0), N = 18	341 (3.1) (0-133)
**ACS cohorts**	24	2304 (13-1127)	43.2 (30.9-52.8), N = 21	1339 (66.2) (10-886), N = 14	13.5 (0.01-20), N = 7 (8.4) (0.01-9.7), N = 4	167 (7.2) [0-74)
**AA**	7	312 (17-93)	41.8 (35.5-52.8), N = 7	195 (80.9) (13-85), N = 4	13.4 (0.01-16.8), N = 2 (8.4) (0.01-3.1), N = 2	14 (4.5) (0-5)
**BAH**	2	58 (13-45)	34.1 (30.9-35), N = 2	47 (65.7) (10-37), N = 5	16.8, N = 1	11 (19.0) (4-7)
**Combined ACS**	15	1934 (14-1127)	43.8 (31.0-49.0), N = 12	1097 (63.9) (12-886), N = 8	13.4 (4.0-20.0), N = 4 (8.4) (5.0-9.7), N = 2	142 (6.2) (0-74)
**Combined types of CS cohorts**	19	1906 (15-473)	38.7 (28.0-50.6), N = 13	877 (56.6) (9-363), N = 10	6.2 (0.02-12.1), N = 8 (8.4) (4.0-6.7), N = 4	131 (6.9) (0-74)

Abbreviations: AA, adrenal adenoma; ACS, adrenal Cushing syndrome; BAH, bilateral adrenal hyperplasia; CD, Cushing disease; CS, Cushing syndrome; NA, not applicable.

^*a*^Weighted by total patients.

In terms of risk bias, using the modified ROBINS-I tool reported earlier, 43% of the studies had low risk, 45% moderate, 2% serious, and in 10%, the assessment was inconclusive.

### Pooled Standardized Mortality Ratio of Cushing Syndrome and Subtypes

**Figure 2.**
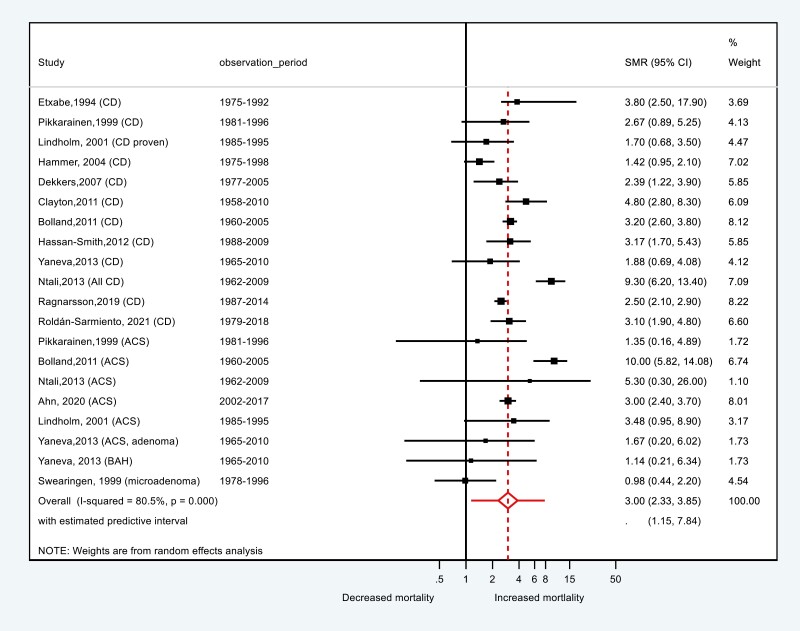
Forest plot presenting standardized mortality ratio (SMR) all-cause mortality of all types of Cushing syndrome. ACS, adrenal Cushing syndrome; BAH, bilateral adrenal hyperplasia; CD, Cushing disease.

**Figure 3.**
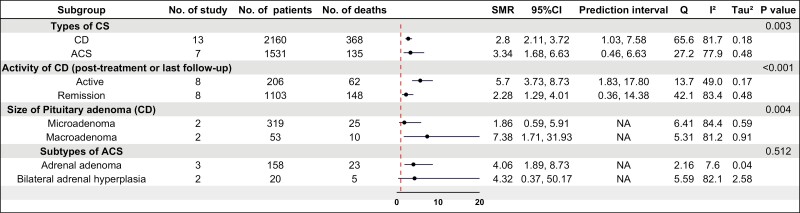
Forest plot of standardized mortality ratio stratified by subgroups of Cushing syndrome (CS) and factors contributing to mortality. The heterogeneity between subgroups analyzed by metan package being taken only from the inverse-variance fixed-effect model. AA, adrenal adenoma; ACS, adrenal Cushing syndrome; BAH, bilateral adrenal hyperplasia; CD, Cushing disease; NA, not applicable.

The pooled SMR for all CD and ACS was 3.00 (95% CI, 2.33-3.85) with an estimated predictive interval of 1.2-7.8 (Figs. 2 and 3). For CD, SMR was 2.8 (95% CI, 2.1-3.7), which was significantly lower than for ACS (SMR = 3.34; 95% CI, 1.68-6.63; *P* < .01).

SMR was higher in patients with active disease (SMR = 5.7) than in those in remission (SMR = 2.3, *P* < .001) and in CD patients with pituitary macroadenomas (SMR = 7.4) compared to microadenomas (SMR = 1.9, *P* < .01) (see Fig. 3). Estimates for men and women were similar.

### Meta-regression Analysis for Proportions of Deaths in Cushing Syndrome and Subtypes

The overall proportion of deaths in CS derived from meta-regression was 0.05 (95% CI, 0.03-0.07). The proportion of deaths adjusted by CS subtype was 0.04 (95% CI, 0.03-0.06) for CD, 0.02 (95% CI, 0.01-0.05) for AA, 0.09 (95% CI, 0.03-0.26) for BAH, and 0.08 (95% CI, 0.04-0.15) for combined ACS.

### Mortality Trends Over the Period of Study

The analyzed articles changed with time, moving from a predominance of adrenal-based CS to CD and subtypes of ACS in later years, especially post 2000. The discoveries of modern diagnostic testing provide an early and accurate diagnosis; surgical methods and medical treatment also advance; all of these are supported by the volume and information of publications, particularly after 2000. For the reasons stated previously and because of the availability of data, the year 2000 was chosen to investigate the difference in mortality in endogenous CS. However, the analysis of SMR articles found that publication before and after 2000 did not represent the SMR between 2 periods because of the overlap of patients; SMR publications post 2000 involved patients with the disease before 2000 (see Table 2).

**Figure 4.**
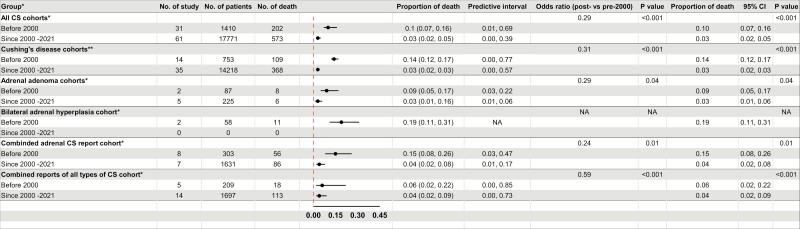
Estimates of the proportion of deaths in subtypes of Cushing syndrome (CS), stratified by published year (before **2000** vs after **2000).** NA, not applicable. *, random-effects logistic regression fitted with period of study; ** fixed-effects logistic regression fitted with period of study.

The proportion of deaths was 0.10 (10%) for studies published pre 2000 compared to 0.03 (3%) since 2000 until 2021 (*P* < .001), with an overall 71% decrease in the proportion of deaths over time (Fig. 4). These findings were consistent (for all subtypes of CS (*P* < .05) except BAH, for which there were insufficient publications after 2000. This finding might relate to a reduction in the overall proportion of deaths during the perioperative period (first 30 days post surgery) which decreased, from 0.04 (95% CI, 0.02-0.09) for articles before 2000 to 0.01 (95% CI, 0.00-0.01) post 2000 (*P* < .01).

### Causes of Death

**Figure 5.**
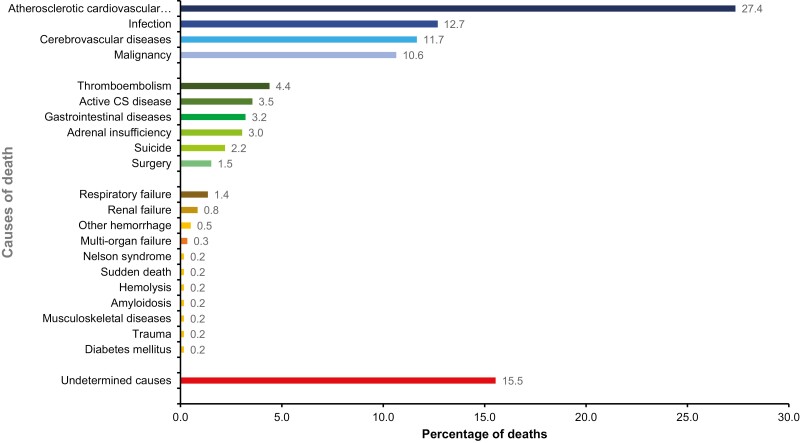
Causes of death from all Cushing syndrome (CS) cohorts (n = 64**).**

The causes of death in CS were reported in 68 study cohorts and included 592 deaths of 7255 patients (Fig. 5). The major contributor to excess mortality was CVD (comprising atherosclerotic heart disease, cerebrovascular disease, and venous thromboembolism [VTE]), which resulted in 43.4%, 43.7%, and 38.6% of total deaths for all CS, CD, and ACS, respectively. The second most common cause of death was infection in 12.7%, 11.5%, and 15.9% of all CS, CD, and ACS cohorts. Analysis of SMR were consistent with estimates of 5.53 (95% CI, 2.51-12.21; *I*^2^ = 81.5%) for CVD and 8.5 (95% CI, 1.65-43.42; *I*^2^ = 36.1%) for infection. Malignancy was the third most common cause for death. The etiology of malignancy was known for only 18 of 55 cases: 6 were pituitary carcinomas, 5 pulmonary malignancies, 3 colon or rectal cancers, and 1 each for prostate, uterus, pancreas, and medullary thyroid cancer. Undetermined causes of death accounted for 15% of deaths.

## Discussion

This is the largest and the most inclusive systematic review and meta-analysis of mortality undertaken in the real-world CS population. The results from this analysis of 3691 patients from 20 studies extend the findings of a previous report, which included 7 research studies comprising 779 patients with CS from 1994 to 2007 (17). Pooled SMR for all subtypes of CS was 3.0 and was significantly higher in ACS compared with CD patients (3.3 vs 2.8; *P* < .01).

The evidence was more substantial than the previous and more restricted study; conducted almost 10 years later, we demonstrated higher SMR across all subtypes of CS. Patients with active disease had a statistically significantly higher SMR than those in remission (5.7 vs 2.3; *P* = .001) with a median follow-up ranging from 7.5 to 15 years. Despite being in remission, mortality was still 2.3 times higher than an age- and sex-matched general population. The results corroborated a previous meta-analysis of mortality in CD patients that included 8 cohort studies with 766 patients and revealed a pooled SMR of 2.5 (95% CI, 1.4-4.2) in treated CD patients, compared to an SMR of 4.6 (95% CI, 2.9-7.3) for patients who remained uncured following transsphenoidal surgery (44). A further multicenter study indicated increased mortality in CD with an overall SMR of 1.6 after more than 10 years of remission thought to be due to increased rates of diabetes mellitus (DM) and cardiovascular complications (45). Our data did not allow us to examine disease duration before diagnosis, disease severity, comorbidities, or the duration of hypercortisolemia, which is critical for considering and minimizing poor long-term outcomes. Indeed, it is highly likely that irreversible effects of prior CS, even in remission, result in increased mortality, with the gap between disease presentation and diagnosis estimated to be over 3.5 years in the ERCUSYN cohort (24). This is supported by a 6.5-year follow-up of CS in New Zealand; despite 80% to 90% of patients undergoing remission, mental illness, osteoporosis, dyslipidemia, and type 2 DM were not reversible after treatment (9). Furthermore, ischemic heart disease, cerebrovascular disease, and hypopituitarism deteriorated throughout the follow-up period (9). Nevertheless, our findings of much higher SMR in active or persistent disease further emphasizes the need for rapid diagnosis and effective therapies to normalize hypercortisolism. Our meta-analysis also demonstrated a higher SMR in patients with macroadenoma vs microadenoma (7.4 vs 1.9, *P* < .01). This is perhaps not surprising based on meta-analyses of outcomes of pituitary surgery for CD, in which microadenomas are more likely to enter remission than macroadenomas (83% *vs* 68%, *P* < .01) (46).

It was not possible to estimate the proportion of deaths in relation to disease activity in CS because of the substantial heterogeneity across studies (eg, differences in inclusion, study periods [1962-2018], regional guideline implementation, laboratory tests, and standard reference ranges).

Few papers have reported SMR estimates of ACS compared to other subtypes of CD. A previous meta-analysis reported an SMR of 1.90 (95% CI, 0.93-3.91; 72 patients) for AA. Our study estimated an SMR of 3.34 (95% CI, 1.68-6.63; 1531 patients) for combined ACS, 4.06 (95% CI, 1.89-8.73; 158 patients) for AA, and 4.32 (95% CI, 0.37-50.17; 20 patients) for BAH. Differences in risk estimates might be explained by 1) the risks of events and time to event outcomes; 2) the period of patient enrollment, mostly before 1990 for ACS and involved pre computed tomography, pre magnetic resonance imaging, and pre transsphenoidal surgery (6, 9, 12, 13, 42), which may contribute to higher mortality. The 95% CIs in all subgroups of adrenal CS were wider than for CD, most likely explained by reduced sample sizes.

Our research enabled assessing changes in CS mortality over time. The overall proportion of deaths significantly decreased post 2000 compared to pre 2000 (3% vs 10%, *P* < .001) and consistent results were found for CD, AA, and combined ACS. Surgery is the mainstay of the initial treatment of CS. Our findings suggest that the proportion of deaths was higher following adrenalectomy for CD or ACS (3% or 4%) compared to transsphenoidal surgery for CD (1%). These findings should be taken cautiously since the introduction of transsphenoidal surgery for pituitary adenoma began in the mid-1970s to the late 1990s. Historically, CS was diagnosed clinically and treated with bilateral adrenalectomy. Secondly, adrenalectomy in CD can be a late treatment option for severe or life-threatening CS. A decrease in perioperative deaths was also demonstrated after 2000 for all CS and combined ACS cohorts. Improved surgical techniques have played an important role in improving operative outcomes, notably transsphenoidal surgery for CD. Laparoscopic adrenal surgery for ACS was regularly performed only in the early to mid 1990s, becoming properly widespread in the 2000s.

A recent meta-analysis described an odds ratio for VTE in CS compared to a general population of 17.8 (95% CI, 15.2-20.8; *P* < .001); and in CS undergoing surgery without compared with anticoagulant prophylaxis of 0.34 (95% CI, 0.19-0.36; *P* < .001) vs 0.26 (95% CI, 0.07-0.11, *P* < .001), respectively (47). In our study, the VTE death rate for overall CS was 4.4% and comparable between CD (3.4%) and ACS (3.4%); however, in the combined CS cohort this was 10.6%. Prescribing anticoagulants for patients with CS is likely to be beneficial.

Atherosclerotic cardiovascular and cerebrovascular diseases, infection, and malignancy, were the leading causes of death across our study. The causes of death can be compared to the report of CS morbidities in the Swedish National Patient Register with high standardized incidence ratios for VTE of 4.9 (95% CI, 2.6-8.4), stroke of 3.1 (95% CI, 1.8-4.9), and sepsis of 6.0 (95% CI, 3.1-10.6) (48). In comparison, the standardized incidence ratio for myocardial infarction was 3.6-4.4 (95% CI, 1.2-11.4) and was significantly higher in the first 3 years before diagnosis. There is supporting evidence that excess cortisol states increase atherosclerotic risks and pathogenesis of CVD events (49). These include DM and insulin resistance (46), hypertension (50), dyslipidemia (51, 52), reduced coronary flow (53), hypercoagulable states, and arthrofibrosis (54). The increased incidence of infections may be explained by the immunosuppressive effects of hypercortisolism, DM, or hyperglycemia together with vascular insufficiency from CS. The limited amount of detail as it relates to the type of infection/organism or opportunistic infection in our study will hopefully trigger further studies; in the interim empirical treatment, especially in life-threatening situations, should be given to prevent/treat underlying infection.

Of note, suicide accounted for 2.2% of all deaths. Neuropsychiatric manifestations of CS are common and can be debilitating (55). Depression (55%-81%) (56–58), panic attacks (53%) (57), anxiety (12%-79%) (57), sleep disorders, psychosis (8%), and cognitive impairment (57, 59) are all recognized.

Adrenal insufficiency, reported as a cause of death in 3% in our study, is a preventable cause of death if CS patients are treated with adequate glucocorticoid replacement during the postoperative period in the suspected remission phase (60). We were unable to identify the period of event or effect of CS subtype or surgery because of a lack of individual patient data.

Follow-up cohorts between 1 to 10 years did not demonstrate any influence of follow-up on death. However, when the median duration of follow-up was more than 10 years, there was a significantly increased mortality compared to less than 5 years’ follow-up for all CS and CD cohorts. Clearly, 2 periods of mortality affected the frequency of fatalities: within 90 days after treatment and a follow-up of more than 10 years. For early deaths, ERCUSYN showed that 49% of CD or ACS mortality occurred within 90 days after first treatment (24). Interestingly, death was attributed to acute complications, which rose dramatically during the postoperative period, accounting for 60% of total deaths (61). In addition, during the first 90 days of therapy, dynamic changes in cortisol levels from hypercortisolism to adrenal insufficiency (due to hypothalamic-pituitary-adrenal axis suppression or hypopituitarism) were offered as supportive evidence for early mortality. Furthermore, death after 10 years might be explained by the recurrence of CS or morbidities associated with CS through long-term cortisol exposure. The recurrence rate was supported by one-third of CD patients encountering a relapse rate during a 10-year period after surgery: 18% (95% CI, 14%-22%; *P* < .01) of CD had recurrence at 50.3 ± 24.0 months after surgery (range, 3.0-205.0 months), and 28% (95% CI, 16%-42%; *P* < .01), at 17.8 ± 15.0 months after surgery (range, 2.0-76.0 months) (46).

The strength of our study is the reach of what is a worldwide CS database including national publications from the United States, Europe, Denmark, Sweden, Korea, New Zealand, and Mexico. The novelty of this work is the development of a methodological pipeline for the meta-analysis of single-arm proportion data by using the *metapreg* program, with our study being the first use of this comprehensive tool. The assumption from the program was binomial distribution fitted with a generalized linear mixed model (62) with a logit link as recommended by Schwarzer et al (63) and Stijnen et al (64). We propose that this analysis is superior to the classic meta-analysis of proportion data using the inverse variance method or Freeman-Tukey double arcsine transformation (65, 66), which assume data are normally distributed. We also presented the prediction interval together with 95% CI as the index of precision for mean estimates, which was the property of the sample, not the population. Further, we put a great deal of effort into excluding duplicate patient cohorts if the same authors, hospitals, cities, or countries were presented across the whole articles.

All studies of this nature have limitations. Meta-analysis was performed at the study and not the individual patient level, limiting the possibility to explore true differences in subtypes of CS characteristics, confounding factors (especially comorbidity and cointervention), disease activity, recurrence, treatment, and mortality. Selection bias may have influenced results as the review was restricted to articles published in English and peer-reviewed journals. Mortality outcomes may be underestimated if patients died before diagnosis and treatment. The information during the perioperative period was scanty for exploring the prediction of deaths. The causes of death should be interpreted with caution because 15.5% of CS in the articles reported “unknown” as the cause of death, and 219 deaths out of 11 300 CS patients from 25 articles failed to report causes of death. Despite known causes, the real etiology could not be confirmed: for example, organ failure or the consequence of active CS. Sources of death certificates or information were also included in several methods. Our data could not explore the duration of disease before diagnosis, the severity of the diseases, including comorbidities, or the duration of hypercortisolemia, which are all important factors for reducing negative long-term outcomes.

CS treatment guidelines focus on achieving normalization of hypercortisolism, prevention or control of comorbidities, long-term disease monitoring and control without recurrence, and elimination of pituitary compression on the adjacent structures while preserving normal pituitary function in the case of CD. Based on this study, we believe it is timely to also focus on reversal of the unacceptable increase in mortality. Our study supports the recent CD guideline recommendation to prevent complications of CS, especially hypercoagulability, CVD, osteoporosis, and fracture in CD (67). Anticoagulants are suggested for thromboprophylaxis preoperatively (ideally 1*-*2 weeks) and for 1 *to* 2 days of the hospital stay and up to 2 *to* 4 weeks or even longer, postoperatively. Screening for CVD/cerebrovascular disease, risk stratification, and consideration of medical therapy as the bridging therapy for hypercortisolism before surgery are all recommended (67). Here, appropriate cortisol replacement therapy for adrenal insufficiency and infection prophylaxis should also be considered. The early detection of recurrence of CS highlights the need for long-lasting follow*-*up of patients with CS. Our data suggest that aggressive cardiometabolic management may benefit at least 50*%* of CS patients and improve long-term mortality. A concerning issue is the risk of suicide, and psychological evaluation may improve the management of all CS patients.

## Data Availability

All data generated or analyzed during this study are included in this published article or in the data repositories listed in “References” (68).

## Abbreviations

AA: adrenal adenoma
ACS: adrenal Cushing syndrome
ACTH: adrenocorticotropin
BAH: bilateral adrenal hyperplasia
CD: Cushing disease
CS: Cushing syndrome
CVD: cardiovascular disease
DM: diabetes mellitus
SMR: standardized mortality ratio
VTE: venous thromboembolism
